# Ethical Parables

**Published:** 1920-09

**Authors:** George Wood Clapp


### ETHICAL PARABLES

BY GEORGE WOOD CLAPP, D.D.S.

A philosopher once determined to seek a practical application of the term "professional ethics." "I will go among the dentists," said he. "They have talked and written much of it. They should know."

He came first to the office of a dentist who was working in the evening. "I seek the practical meaning of the words 'professional ethics,'" said he, "and the manner of its application."

"I can tell you," said the dentist, "for I am strictly ethical. It is to do one's best for one's patients, at fees they can easily pay."

"That is certainly a noble object," said the philosopher, "and I am sure you must be happy in achieving it. Surely your grateful patients must take as great pains to pay as much as they can afford as you take to serve them as well as you can. And, of course, they pay you promptly."

"I have not noticed any eagerness on their part to pay all they can afford," replied the dentist. "Some do not pay promptly and a few for whom I have done my best have never paid. I have to work hard all day and many evenings. Even then I find it difficult to keep my head above the financial waves, and can not give my family many of the comforts to which they are entitled. Luxuries are few at our house. I doubt whether I am nearly as well off financially as the average of those I serve."

"Alas," said the philosopher, "I see that you know neither the definition of ethics nor the manner of its application. You consider only one party to the transaction, the patient, and leave yourself and those dependent upon you to suffer the fruits of your own injustice."—*Dental Digest.*

